# Prevalence and Clinical Significance of Residual or Reconverted Red Bone Marrow on Knee MRI

**DOI:** 10.3390/diagnostics11091531

**Published:** 2021-08-25

**Authors:** Minh Tu Vo, Ambrish Singh, Tao Meng, Jasveen Kaur, Alison Venn, Flavia Cicuttini, Lyn March, Marita Cross, Terence Dwyer, Andrew Halliday, Graeme Jones, Changhai Ding, Benny Antony

**Affiliations:** 1 Menzies Institute for Medical Research, University of Tasmania, Private Bag 23, Hobart, TAS 7000, Australia; mtvo@utas.edu.au (M.T.V.); ambrishagastya@gmail.com (A.S.); tao.meng@utas.edu.au (T.M.); jasveen.kaur@utas.edu.au (J.K.); alison.venn@utas.edu.au (A.V.); terence.dwyer@wrh.ox.ac.uk (T.D.); graeme.jones@utas.edu.au (G.J.); changhai.ding@utas.edu.au (C.D.); 2 Department of Epidemiology and Preventive Medicine, Monash University, Melbourne, VIC 3004, Australia; flavia.cicuttini@monash.edu; 3 Institute of Bone and Joint Research, University of Sydney, Sydney, NSW 2065, Australia; lyn.march@sydney.edu.au (L.M.); maritac@med.usyd.edu.au (M.C.); 4 The George Institute for Global Health, Nuffield Department of Obstetrics & Gynaecology, University of Oxford, Oxford OX3 9DU, UK; 5 Department of Radiology, Royal Hobart Hospital, Hobart, TAS 7000, Australia; andrew.halliday@ths.tas.gov.au; 6 Clinical Research Centre, Zhujiang Hospital of Southern Medical University, Guangzhou 510280, China

**Keywords:** residual/reconverted red bone marrow, young adults, knee pain, magnetic resonance imaging

## Abstract

Background: Residual/reconverted red bone marrow (RBM) in adult knees is occasionally observed on routine knee magnetic resonance imaging (MRI). We aimed to identify its prevalence, distribution, and associations with lifestyle factors, knee structural abnormalities, and knee symptoms in young adults. Methods: Participants (*n* = 327; aged = 31–41 years) were selected from the Childhood Determinants of Adult Health (CDAH) knee study. They underwent T1-weighted and proton-density-weighted fat-suppressed MRI scans of knees. Residual/reconverted RBM in distal femur and proximal tibia were graded semi-quantitatively (grades: 0–3) based on the percentage area occupied. Knee structural abnormalities were graded semi-quantitatively using previously published MRI scoring systems. Knee symptoms (pain, stiffness, and dysfunction) were assessed using the Western Ontario and McMaster Universities Osteoarthritis Index (WOMAC) scale during CDAH knee study (year: 2008–2010) and at 6–9-year follow-up during the CDAH-3 study (year: 2014–2019). Associations between definite RBM (grade ≥ 2) and lifestyle factors, knee symptoms, and structural abnormalities were described using log-binomial regressions. Results: Definite RBM was seen in females only, in 29 out of 154 cases (18.8%), with femoral involvement preceding tibial involvement. Definite RBM was associated with increased BMI (PR = 1.09/kg/m^2^; 95% CI: 1.03, 1.16), overweight status (PR = 2.19; 95% CI: 1.07, 4.51), and WOMAC knee pain (PR = 1.75; 95% CI: 1.11, 2.74) in cross-section analysis. However, there was no association between RBM and knee-pain after seven years (PR = 1.15; 95% CI: 0.66, 2.00). There were no associations between RBM and knee structural abnormalities. Conclusion: Presence of definite RBM in young adult knees was observed in females only. Definite RBM was associated with overweight measures, and the modest association with knee pain may not be causally related.

## 1. Introduction

Bone marrow is broadly classified into red and yellow subtypes based on macroscopic appearance. Red bone marrow (RBM) is haematopoietically active, abundantly vascularized, and has high erythrocyte cellularity [1,2,3]. In contrast, yellow bone marrow (YBM) is haematopoietically inactive, scantily vascularised, and has high adipocyte cellularity [1,2]. RBM is chemically composed of 40% fat, 40% water, and 20% protein, whereas YBM is composed of 80% fat, 15% water, and 5% protein [4,5]. These differences in chemical composition give RBM and YBM different appearances on magnetic resonance imaging (MRI) [6,7,8,9,10]. RBM appears hypointense compared to skeletal muscles on T1-weighted MRI, while YBM appears hyperintense. RBM is hyperintense to YBM on a proton-density-weighted fat-suppressed MRI.

Infants are born with RBM in their entire skeleton, but RBM in their appendicular skeleton gradually converts to YBM with age [5,11,12,13,14]. This physiological process begins in the terminal phalanges and progresses from distal to proximal. Within each long bone, the conversion first occurs in the epiphysis, then diaphysis, and later from the diaphysis towards the metaphyses. Marrow conversion is completed by 24 years of age, at which time RBM is confined to the axial skeleton, plus the proximal femora and humeri [15,16]. At 25 years of age, knees commonly contain YBM only. 

Nonetheless, RBM is occasionally observed in adult knees on a routine MRI. First documented in obese females, RBM in adult knees was thought to be of malignant myeloproliferative origin, but further investigations showed otherwise [17]. RBM was subsequently observed in the knees of healthy adults, with a high prevalence among marathon runners [18]. A high prevalence of RBM was also observed in the knees of heavy smokers, without associated symptomatology [19]. A survey of RBM in the knees of healthy adults concluded that RBM was a common benign finding [20]. RBM proliferation in the knees of healthy adults in the absence of a malignant cause is postulated to be either a naturally occurring biological variation [21] or an innocuous physiological response to hematopoietic stress, such as sports anaemia [8,18]. There are conflicting reports on the associations between RBM in adult knees with haemoglobin levels and anaemia [22,23]. Nonetheless, RBM in such cases is unrelated to infiltrative marrow pathology [24] and is referred to as residual or reconverted RBM [22,23].

Residual or reconverted RBM has not been studied extensively. Previous studies documenting the prevalence of residual or reconverted RBM relied on limited sample sizes (*n* = 190 at best), which were prone to selection bias. Additionally, residual or reconverted RBM distribution patterns within the knee joint among the distal femur, proximal tibia, and proximal fibula were poorly documented. Moreover, associations between residual or reconverted RBM and clinical symptoms and knee structural abnormalities have not been examined. This study aimed to describe the prevalence and pattern of distribution of residual or reconverted RBM in a population-based sample of Australian young adults, and to describe its cross-sectional associations with lifestyle factors, structural abnormalities, and knee symptoms, as well as its longitudinal association with knee symptoms. We hypothesized that residual or reconverted RBM is distributed more prevalently in the knee MRI of young female adults.

## 2. Methods

### 2.1. Study Design

This study was a descriptive study based on the data collected from an observational cohort. This report follows the Strengthening the Reporting of Observational Studies in Epidemiology (STROBE) Statement guidelines [25].

### 2.2. Participants

Participants (*n* = 327, aged 31–41 years) were selected from the Childhood Determinants of Adult Health (CDAH) knee study during 2008–2010. The CDAH knee study was a follow up on a subsample (*n* = 330, aged 31–41 years) of participants in the CDAH study conducted during 2004–2006 [26,27]. The CDAH study was a follow up on a subsample (*n* = 2410, aged 26–36 years) of an Australia-wide sample of young adults who were part of the Australian Schools Health and Fitness Survey (ASHFS) (*n* = 8498, aged 7–15 years) conducted in 1986. During 2008–2010, CDAH participants who resided in metropolitan Melbourne and Sydney (*n* = 764) were invited to participate in the CDAH knee study. Those who accepted the invitation (*n* = 529) were assessed against exclusion criteria of being pregnant; history of knee cartilage diseases, such as rheumatoid arthritis; or any contraindication for MRI. Those eligible (*n* = 449) were asked to complete a short computer-assisted telephone interview and undergo a knee MRI scan at Epworth Hospital in Melbourne or at North Shore Private Hospital in Sydney. Some participants (*n* = 119) did not undergo MRI due to long distances to the hospital, work or family commitments, moving interstate, becoming pregnant, or changing their mind. Three unreadable MRI scans were excluded, and the remaining participants (*n* = 327) were included in this study (Supplemental Figure S1). For the longitudinal analysis with knee symptoms, participants were selected for whom MRI scans were available from the CDAH knee study and were followed up with in the CDAH-3 study after 6–9 years (year: 2014–2019).

This study was approved by the Southern Tasmania Health and Medical Human Research Ethics Committee, the Monash University Human Research Ethics Committee, and the Northern Sydney and Central Coast Area Human Research Ethics Committee. All participants provided written informed consent.

### 2.3. Demographics and Lifestyle Factors

Weight and height were measured to the nearest 0.1 unit (kg and cm, respectively) with shoes, socks, and bulky clothing removed. Body mass index (BMI) was calculated as weight over height squared, with overweight status as BMI > 25 kg/m^2^. Smoking status was defined by whether a subject was an active smoker in the CDAH study. History of knee injury in childhood and adulthood requiring surgery or non-weight-bearing treatment for more than 24 h were assessed in the CDAH knee study. Total physical activity was measured in hours per week using the long version of the International Physical Activity Questionnaire (IPAQ), which has acceptable reliability (ICC = 0.64; 0.55–0.72) [28] and validity (ICC = 0.638) [29] [Cronbach’s alpha 0.769–1.00] [30,31]. 

### 2.4. Knee Symptom Measurements

Knee symptoms were assessed using the Western Ontario and McMaster Universities Osteoarthritis Index (WOMAC) questionnaire in the CDAH knee study through a computer-assisted phone interview and via teleform questionnaires for the CDAH-3 study. Participants were asked about their knee pain (5 questions), stiffness (2 questions), and dysfunction (17 questions) status during the past 30 days. Each question was graded on a scale of 0–9, where 0 indicated no symptoms and 9 indicated symptoms of maximum intensity. Total WOMAC scores were calculated by adding the scores of five subscales in WOMAC knee pain, two subscales in WOMAC stiffness, and 17 subscales in WOMAC dysfunction. Presence of any pain, stiffness, and dysfunction was defined as any score ≥1. The WOMAC questionnaire was chosen for its responsiveness to knee symptoms in a young population [32] as well as its tested validity (Cronbach alpha = 0.84–0.95) and reliability (ICC = 0.77–0.89) [33,34].

### 2.5. MRI Measurements

MRI scans were obtained from two centres, which used General Electric Medical Systems, Milwaukee, WI, USA. Knees were imaged in the sagittal plane on a 1.5 T whole-body MRI unit with the use of a commercial transmit–receive extremity coil. Details of the image sequences used are as follows: (1) T1-weighted fat saturation three-dimensional spoiled gradient recall acquisition in the steady-state; flip angle, 55 degrees; repetition time, 58 msecs; echo time, 12 msec; field of view, 16 cm; 60 partitions; 512 × 512 matrix; acquisition time, 11 min 56 sec; one acquisition; sagittal plane; partition thickness, 1.5 mm; in-plane resolution, 0.31 × 0.31 (512 × 512 pixels). (2) Proton-density-weighted fat-saturated two-dimensional fast spin-echo; coronal plane; partition thickness, 3.3 mm; in-plane resolution, 0.31 × 0.31 (512 × 512 pixels) [35]. 

**Figure 1:**
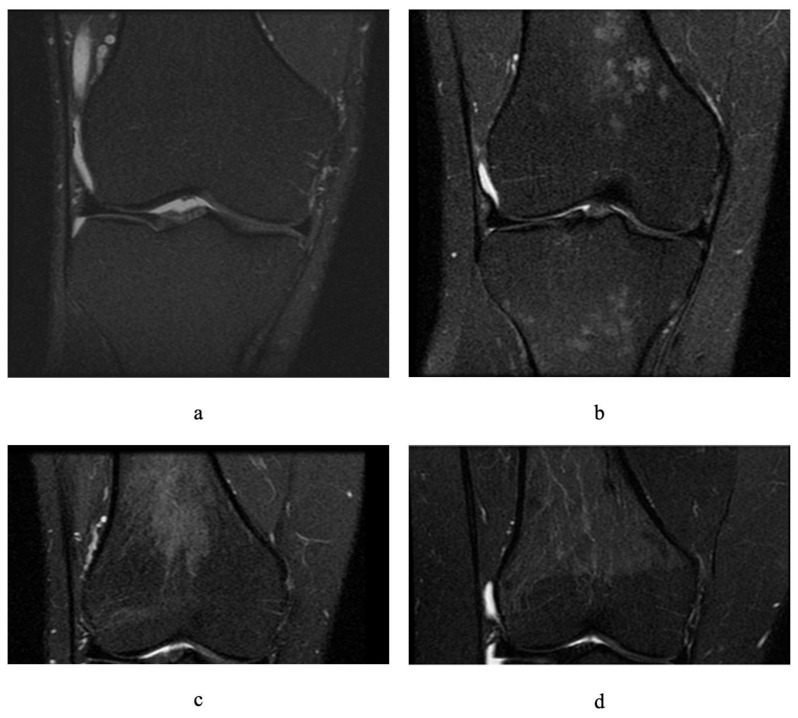
Different grades of residual or reconverted red bone marrow on fat-saturated proton-density-weighted magnetic resonance images of adult knees (coronal sections): (**a**) grade 0 in femur and tibia, (**b**) grade 1 in femur and tibia, (**c**) grade 2 in femur, (**d**) grade 3 in femur.

Residual or reconverted RBM in the knee was assessed on fat-saturated proton-density-weighted MRI by identifying foci of hypointensity above the distal femoral epiphyseal plate and below the proximal tibial plate. RBM in the femur and tibia were graded on a scale of 0–3 using similar criteria as those in a previous study [22], where grade 0 indicated 0% occupancy of the metaphysis, grade 1 indicated >0 to <30% occupancy of the metaphysis, grade 2 indicated 30 to 60% occupancy, and grade 3 indicated >60% occupancy (Figure 1). Due to the fact that some presence of grade 1 RBM could be considered a biological variation or doubtful RBM [21], definite residual or reconverted RBM was defined as grade ≥ 2 in either the femur or the tibia. RBM assessment was performed by a medical student (Jasveen, K) under the supervision of an experienced radiologist (Halliday, A) to exclude any pathological lesion. The intra-rater ICC was estimated as 0.91; 95% CI 0.84–0.95 and inter-rater ICC was 0.94; 95% CI: 0.89, 0.97 (calculated using Stata 15 based on a single measurement, absolute agreement, 2-way mixed effects model). 

Cartilage defects, subchondral bone marrow lesions (BMLs), meniscal tears, and meniscal extrusions in the knee were assessed in the CDAH knee study as published previously [35]. BMLs were graded on a scale of 0–3, based on coronal proton-density-weighted images based on the percentage of BML occupancy, where grade 0 indicated 0% of the area, grade 1 indicated ≤30% of the area, grade 2 indicated >30% to <60% of the area, and grade 3 indicated >60% of the area. The presence of BML was defined as grade ≥ 1 in either the femur or tibia. Cartilage defects were graded on a scale of 0–4 on T1-weighted fat-saturated sagittal images and proton-density-weighted coronal images, where grade 0 indicated normal cartilage, grade 1 indicated focal blistering, grade 2 indicated a loss of thickness of less than 50%, grade 3 indicated a deep ulceration with loss of thickness > 50%, and grade 4 indicated a full-thickness chondral wear with exposure of subchondral bone. The presence of a cartilage defect was defined as grade ≥ 2 in either the femur or tibia. Meniscal tear was graded on a scale of 0–2 based on proton-density-weighted coronal and T1-weighted sagittal images using a combined whole-organ magnetic resonance imaging score (WORMS) scoring system, where grade 0 indicated normal intact meniscus, grade 1 indicated a nondisplaced tear, and grade 2 indicated a displaced tear or maceration. Meniscal extrusion was graded on a scale of 0–2 based on the proton-density-weighted coronal images, where grade 0 indicated intact meniscus, grade 1 indicated a partially displaced meniscus with respect to tibia, and grade 2 indicated a completely displaced meniscus. The presence of meniscal tears and meniscal extrusions in the knee were defined as grade ≥ 1 in either the femur or tibia.

### 2.6. Statistical Analysis

The prevalence of RBM in the knee was calculated from the number of participants with definite RBM (grade ≥ 2). Log-binomial regressions were used to describe the cross-sectional associations between the presence of definite RBM in the knee and lifestyle factors (overweight status, smoking status, and total physical activity), knee symptoms (total WOMAC pain, stiffness and dysfunction scores), and knee structural abnormalities (cartilage defect, subchondral BMLs, meniscal tear, and meniscal extrusion) and longitudinal association between RBM and knee symptoms. Age, BMI, and previous knee injuries were included as potential confounders. All statistical analyses were performed using Stata 16 (Stata Corp, College Station, TX, USA). 

## 3. Results

### 3.1. Prevalence

Graded measurements of residual or reconverted RBM reported by sex and by location within the knee joint are shown in Table 1. The prevalence of RBM in the knee was calculated from the number of participants with a definite RBM (grade ≥ 2). In male participants, definite residual or reconverted RBM was not observed. Therefore, male participants were excluded from further analyses. In female participants, definite RBM was observed in 18.8% (*n* = 29) of femora and 3.9% (*n* = 6) of tibiae. Both grade 2 (*n* = 23) and grade 3 (*n* = 6) RBM were observed in the femora. Only grade 2 RBM was observed in the tibiae. 

### 3.2. Pattern of Distribution

**Figure 2:**
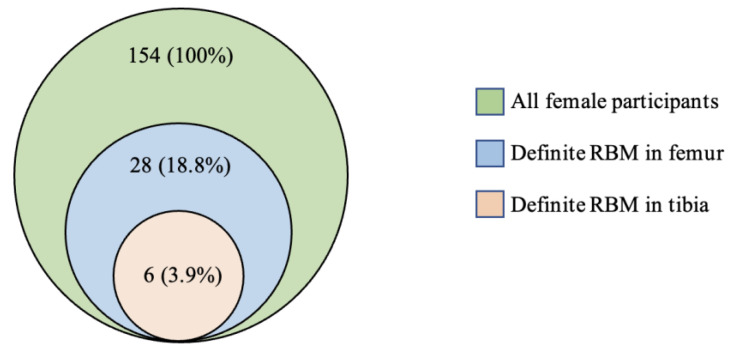
Co-occurrence of residual or reconverted red bone marrow in the femur and tibia in young female adults.

The co-occurrences of residual or reconverted RBM in the femur and tibia of female participants are shown in Figure 2. Definite RBM (grade ≥ 2) in the tibia was only observed alongside concomitant definite RBM in the femur. 

### 3.3. Associations with Lifestyle Factors

Characteristics of female participants based on the prevalence of residual or reconverted RBM are shown in Table 2. Female participants who had definite RBM had higher overweight status, mean BMI, and WOMAC pain than female participants with no definite RBM. There was no significant difference in age, past knee injury, smoking status, weight, total physical activity, WOMAC stiffness, WOMAC dysfunction, or knee structural abnormalities between female participants with and without definite RBM. 

Cross-sectional associations of residual or reconverted RBM with demographics and lifestyle factors in female participants are shown in Table 3. Definite RBM was associated with weight, overweight status, and BMI in both univariable and multivariable analyses. Residual or reconverted RBM was not associated with age, past knee injury, smoking status, or total physical activity in either univariable or multivariable analysis. 

### 3.4. Associations with Knee Symptoms

**Figure 3:**
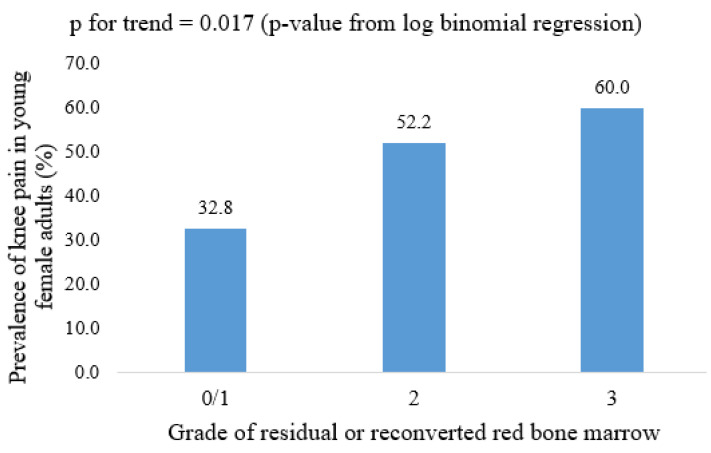
Prevalence of knee pain in different grades of residual or reconverted red bone marrow in young female adults; *p*-value from log-binomial regression.

The cross-sectional and longitudinal associations of a definite residual or reconverted RBM with knee symptoms in female participants are shown in Table 4. Definite RBM was associated with WOMAC pain in both univariable and multivariable cross-sectional analyses. This result remained statistically significant after further adjusting for MRI structural abnormalities, specifically cartilage defects, BMLs, meniscal tears, and meniscal extrusions. However, no significant association was observed between RBM and knee symptoms measured after seven years at CDAH-3. The prevalence of knee pain in different grades of residual or reconverted RBM is shown in Figure 3. Residual or reconverted RBM was not associated with WOMAC stiffness or WOMAC dysfunction in either univariable or multivariable analysis. 

### 3.5. Associations with Degenerative Knee Structural Abnormalities

Cross-sectional associations of residual or reconverted RBM with knee structural abnormalities in female participants are shown in Table 5. Definite RBM was not associated with cartilage defects, subchondral BMLs, meniscal tears, or meniscal extrusions. 

## 4. Discussion

This is the first study to describe the prevalence and pattern of distribution of residual or reconverted RBM in the knee in a population-based sample of Australian young adults. We found the prevalence of definite RBM to be 18.8% in female participants and 0% in male participants. Residual or reconverted RBM always involved the distal femoral region and was associated with overweight measures and knee pain in cross-sectional analysis. However, no significant association was observed between RBM and knee pain in the longitudinal analysis.

The prevalence of residual or reconverted RBM had been previously reported to be 56% in females and 16% in males in one study [20] and 35% overall in another [21]. However, said studies relied on smaller sample sizes selected from hospital patients (*n* = 199 and *n* = 51 respectively) of a wider age range (16–79 and 25–67 years respectively) and did not utilize any (semi-)quantitative scale to grade residual or reconverted RBM. We used a semi-quantitative scoring, following a similar criteria to a more recent study [22], on a population-based sample of largely healthy young adults. We found the prevalence of a definite residual or reconverted RBM (18.8%) to be lower than the prevalence reported previously. Our results were consistent with a more recent study, which reported the prevalence of RBM proliferation to be as low as 20.7% in females and 0% in males, estimated from a smaller sample (*n* = 140) than ours, which was recruited from a single centre in Erzincan [22]. 

The distribution pattern of RBM in adult knees observed in our study was that tibial involvement was only present with concomitant femoral involvement. This was consistent with a previous report, which found that isolated residual or reconverted RBM in the tibia were rare [20]. Our study substantiated such findings with a larger population-based sample of young adults.

There were associations between residual and reconverted RBM and increased weight, overweight status, and BMI in female participants. These findings were consistent with earlier reports of residual or reconverted RBM being common in obese females [17,20,23]. We did not find an association between residual and reconverted RBM and smoking, in contrast to previous reports [19,20]. This could be attributed to the fact that our sample was young, and the cumulative effects of smoking may not yet be present. A recent study that used a semi-quantitative approach to RBM measurement reported the same finding [23]. We did not find an association between RBM and physical activity, whereas a previous study reported increased RBM with vigorous anaerobic exercise [18]. Again, this finding might be attributed to the age and the population-representative nature of our sample. 

Although we observed an association between RBM and knee pain in the cross-sectional analysis, no significant association was observed with knee pain assessed after seven years. The absence of longitudinal association may indicate that the association between RBM and knee pain is not causally related. The clinical significance of residual or reconverted RBM in adult knees was, historically, a differential of myeloproliferative malignancy, as RBM can take on a similar appearance to a pernicious pathological process [2,8,17,21]. More recent studies have examined the relationship between residual or reconverted RBM in adult knees with anaemia. This relationship is complex, as one study found increasing RBM grades to be correlated with an increasing rate and severity of anaemia [23], whereas another found no association between RBM grade and haemoglobin level [22]. We did not find any association of residual or reconverted RBM with knee structural abnormalities and incident knee symptoms. 

The strength of this study is the use of a population-based young adult sample, which was larger than previous studies, hence less susceptible to selection bias. Both cross-sectional and longitudinal analyses were performed to study the association of residual or reconverted RBM with knee symptoms. Nonetheless, the study had several limitations. Our sample was recruited from two metropolitan areas only, and the sample size was not calculated a priori for this analysis. We did not exclude or have information on any thalassemia trait in our participants. Finally, this study did not collect data to elucidate further the relationship between RBM and anaemia, which is still a point of contention in the aetiology of RBM [22,23].

## 5. Conclusions

In summary, residual or reconverted RBM in the knee was only prevalent in females in our population-based sample of young Australian adults. Femoral involvement of RBM preceded tibial involvement. BMI and overweight status were positively associated with the prevalence of definite RBM in young females. Although a modest association between RBM and knee pain was observed in the cross-sectional analysis, the absence of a longitudinal association with knee pain after seven years rules out a causal relationship.

## Figures and Tables

**Table 1:** Graded measurements of residual or reconverted red bone marrow in young adults.

Male (n = 173)—Prevalence (Definite RBM, Grade ≥ 2) = * 0.0%		
RBM Grade	Femur/*n* (%)	Tibia/*n* (%)
0	170 (98.3%)	173 (100%)
1	3 (1.7%)	0 (0.0%)
2	0 (0.0%)	0 (0.0%)
3	0 (0.0%)	0 (0.0%)
Female (n = 154)—Prevalence (Definite RBM, Grade ≥ 2) = * 18.8%		
**RBM Grade**	**Femur/*n* (%)**	**Tibia/*n* (%)**
0	94 (61.0%)	133 (86.4%)
1	31 (20.1%)	15 (9.7%)
2	23 (14.9%)	6 (3.9%)
3	6 (3.9%)	0 (0.0%)

* Prevalence of RBM is defined with definite RBM (grade ≥ 2).

**Table 2:** Characteristics of young female adults based on the prevalence of residual or reconverted red bone marrow.

Prevalence of Red Bone Marrow		Definite RBM Absent (Grade ≤ 1) *n* = 125	Definite RBM Present (Grade ≥ 2) *n* = 29	*p*-Value ^+^
**Demographic and lifestyle factors**				
Age (years)	mean (s.d.)	35.5 (2.7)	35.1 (2.8)	0.534 ^
Knee injury (yes)	*n* (%)	15 (12.3%)	2 (7.1%)	0.438 *
Smoking status (yes)	*n* (%)	37 (31.6%)	7 (25.0%)	0.493 *
Weight (kg)	mean (s.d.)	66.1 (10.6)	71.5 (19.5)	0.053 ^
Overweight status (yes)	*n* (%)	34 (28.6%)	13 (52.0%)	**0.023** *
BMI (kgm^−2^)	mean (s.d.)	24.1 (3.7)	26.7 (6.8)	**0.008** ^
Total physical activity (hours/week)	mean (s.d.)	10.9 (6.8)	9.4 (6.3)	0.310 ^
**Knee symptoms at CDAH knee study**				
WOMAC pain (yes)	*n* (%)	40 (32.8%)	15 (53.6%)	**0.040** *
WOMAC stiffness (yes)	*n* (%)	30 (24.6%)	11 (39.3%)	0.116 *
WOMAC dysfunction (yes)	*n* (%)	47 (38.5%)	15 (53.6)	0.145 *
**Knee symptoms at CDAH-3 study**				
WOMAC pain (yes)	*n* (%)	31 (43.1%)	10 (55.6%)	0.341 *
WOMAC stiffness (yes)	*n* (%)	26 (35.6%)	10 (52.6%)	0.176 *
WOMAC dysfunction (yes)	*n* (%)	30 (41.1%)	8 (42.1%)	0.937 *
**Knee structural abnormalities**				
Cartilage defect (yes)	*n* (%)	53 (43.4%)	11 (37.9%)	0.589 *
Subchondral BMLs (yes)	*n* (%)	35 (28.0%)	8 (27.6%)	0.964 *
Meniscal tear (yes)	*n* (%)	3 (2.5%)	1 (3.5%)	0.771 *
Meniscal extrusion (yes)	*n* (%)	35 (28.9%)	12 (41.4%)	0.194 *

^+^ Bold indicates statistical significance of *p* < 0.05. ^ Differences between two groups were tested using a two-tailed *t*-test. * Differences between two groups were tested using a chi-squared test.

**Table 3:** Cross-sectional associations of definite residual or reconverted red bone marrow with demographics and lifestyle factors in young female adults.

	Univariable PR (95% CI)	Multivariable * PR (95% CI)
Age (year)	0.96 (0.85 to 1.09)	0.97 (0.85 to 1.11)
Knee injury (yes)	0.60 (0.16 to 2.32)	0.47 (0.20 to 1.12)
Smoking (yes)	0.77 (0.35 to 1.67)	0.76 (0.34 to 1.71)
Weight (kg)	**1.02 (1.00 to 1.04)**	**1.03 (1.01 to 1.06)**
Overweight (yes)	**2.24 (1.10 to 4.53)**	**2.15 (1.06 to 4.38)**
BMI (kg/m^2^)	**1.08 (1.03 to 1.14)**	**1.09 (1.03 to 1.16)**
Total physical activity (hours/week)	0.97 (0.92 to 1.03)	0.97 (0.91 to 1.04)

Results are shown in prevalence ratios (95% confidence interval). Bold indicates statistical significance of *p* < 0.05. * Multivariable analysis adjusted for age, BMI, height (if weight was the predictor), and knee injury, depending on the predictor evaluated in the model.

**Table 4:** Associations of definite residual or reconverted red bone marrow with knee symptoms in young female adults.

	Univariable PR (95% CI)	Multivariable * PR (95% CI)
**Cross-sectional association** Predictor variable: RBM CDAH knee cartilage study (yes/no) Outcome variable: WOMAC at CDAH knee cartilage study (yes/no)		
WOMAC pain (yes)	**1.66 (1.06 to 2.51)**	**1.75 (1.11 to 2.74)**
WOMAC stiffness (yes)	1.60 (0.91 to 2.79)	1.29 (0.64 to 2.58)
WOMAC dysfunction (yes)	1.39 (0.92 to 2.10)	1.33 (0.84 to 2.11)
**Longitudinal association** Predictor variable: RBM at CDAH knee cartilage study (yes/no) Outcome variable: WOMAC at CDAH-3 study (yes/no)		
WOMAC pain (yes)	1.29 (0.79 to 2.11)	1.15 (0.66 to 2.00)
WOMAC stiffness (yes)	1.48 (0.87 to 2.50)	1.40 (0.78 to 2.50)
WOMAC dysfunction (yes)	1.02 (0.56 to 1.86)	0.85 (0.46 to 1.59)

Results are shown in prevalence ratio (PR) (95% confidence interval). Bold indicates statistical significance of *p* < 0.05. * Multivariable analysis adjusted for age, BMI, and knee injury.

**Table 5:** Cross-sectional associations of definite residual or reconverted red bone marrow with knee structural abnormalities in young female adults.

	Univariable PR (95% CI)	Multivariable * PR (95% CI)
Cartilage defect (yes)	0.83 (0.42 to 1.64)	0.71 (0.33 to 1.53)
Subchondral BMLs (yes)	0.98 (0.47 to 2.05)	1.13 (0.51 to 2.51)
Meniscal tear (yes)	1.30 (0.23 to 7.39)	1.80 (0.25 to 12.81)
Meniscal extrusion (yes)	1.55 (0.80 to 2.98)	1.63 (0.80 to 3.34)

Results are shown in prevalence ratios (95% confidence interval). * Multivariable analysis adjusted for age, BMI, and knee injury.

## Data Availability

Data sharing not applicable.

## Supplementary Materials

The following are available online at https://www.mdpi.com/article/10.3390/diagnostics11091531/s1, Supplement Figure S1: Participants flow chart, Supplement Table S1: Longitudinal associations of definite residual or reconverted red bone marrow with incident knee symptoms in young female adults.
