# Supplementary material for: Prevalence and Clinical Significance of Residual or Reconverted Red Bone Marrow on Knee MRI
Source: Diagnostics (Basel). 2021 Aug 25;11(9):1531. doi: 10.3390/diagnostics11091531 (PMC8466981; doi:10.3390/diagnostics11091531)
Supplement: Supplementary file 1 [file diagnostics-11-01531-s001.zip › diagnostics-1294380-supplementary.pdf]

Supplement Figure S1. Participants flow chart.

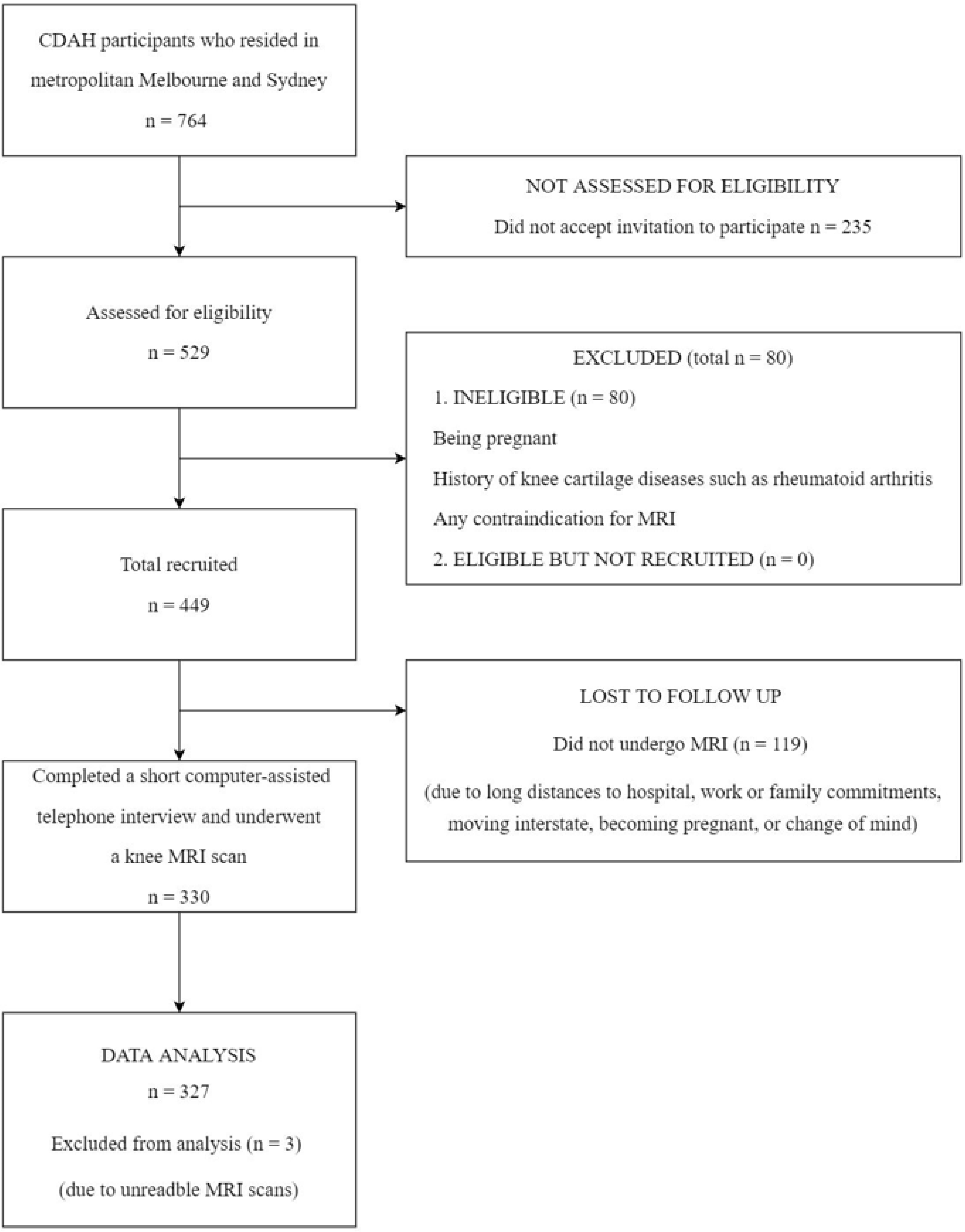

CDAH: Childhood Determinants of Adult Health; MRI: magnetic resonance imaging

Supplement Table S1. Longitudinal associations of definite residual or reconverted red bone marrow with incident knee symptoms in young female adults

	Univariable PR (95% CI)	Multivariable* PR (95% CI)
Predictor variable: RBM at CDAH-knee cartilage study (yes/no)		
Outcome variable: incident WOMAC symptom at CDAH-3 study (yes/no)		
WOMAC pain (yes)	0.40 (0.05 to 2.55)	0.41 (0.06 to 2.59)
WOMAC stiffness (yes)	1.48 (0.64 to 3.40)	1.11 (0.41 to 2.98)
WOMAC dysfunction (yes)	0.59 (0.15 to 2.31)	0.90 (0.26 to 3.19)

Results are shown in prevalence ratio (PR) (95% confidence interval). **Bold** indicates statistical significance of $P < 0.05$. *Multivariable analysis adjusted for age, BMI and knee injury.
